# OCT-Derived Radiomic Features for Differentiating Optic Neuritis Etiologies: An Unsupervised and Supervised Machine-Learning Approach

**DOI:** 10.1016/j.xops.2026.101401

**Published:** 2026-09-07

**Authors:** Hoda El Mohyne, Thierry David, Kevin Mairot, Sarah Demortière, Shailesh Appukuttan, Mario Borrelli, Bertrand Audoin, Jan-Patrick Stellmann, Natacha Stolowy

**Affiliations:** 1 Aix Marseille Université, Centre d'Exploration Métabolique par Résonance Magnétique-Center for Magnetic Resonance in Biology and Medicine, Centre National de la Recherche Scientifique, Marseille, France; 2 Department of Ophthalmology, Assistance Publique Hôpitaux de Marseille, Hopital de la Conception, Marseille France; 3 Aix Marseille Université, Centre National de la Recherche Scientifique, Marseille, France; 4 Aix Marseille Université, Institut de Neurosciences de la Timone, Centre National de la Recherche Scientifique, Marseille, France; 5 Department of Neurology, Assistance Publique Hôpitaux de Marseille, Hopital de la Timone, Marseille, France; 6 Department of Radiology, Assistance Publique Hôpitaux de Marseille, Hopital de la Timone, Marseille, France

**Keywords:** Multiple sclerosis, MOGAD, OCT, Machine learning, Radiomics

## Abstract

**Purpose:**

To assess the feasibility of an OCT-based radiomics approach in optic neuritis (ON) and determine whether peripapillary retinal nerve fiber layer (pRNFL) OCT-derived radiomic features could help differentiate between multiple sclerosis–associated ON (MS-ON) and myelin oligodendrocyte glycoprotein antibody–associated disease ON (MOGAD-ON).

**Design:**

Retrospective cohort study.

**Participants:**

Patients with ON associated with either MS (MS-ON) or MOGAD-ON were selected from the existing Optic Neuropathies of la Timone cohort. In total, 45 patients (61 eyes with ON; 28 with MS and 17 with MOGAD) contributing 266 OCT examinations were included.

**Methods:**

A radiomics pipeline was applied to pRNFL OCT data to extract quantitative imaging features. A total of 102 radiomic features were generated and reduced using feature selection methods. Unsupervised and supervised machine-learning approaches were used to identify relevant radiomic patterns and evaluate their ability to discriminate MS-ON from MOGAD-ON. Models were trained using radiomic features alone, clinical variables alone, and a combination of both.

**Main Outcome Measures:**

The primary outcome measure was the diagnostic performance of machine-learning models in distinguishing MS-ON from MOGAD-ON, assessed using the area under the receiver operating characteristic curve (AUC).

**Results:**

Among the 102 extracted radiomic features, 62 were retained after feature reduction, and 3 features were selected through machine-learning–based analysis. Supervised machine-learning models (logistic regression) using radiomic features outperformed models based on clinical variables alone (AUC, 0.763 vs. 0.584; *P* = 0.001). Combining radiomics with clinical variables yielded a numerically higher AUC of 0.788 than radiomics alone (0.763), but without statistical significance (*P* = 0.361). The combined model remained significantly better than the clinical-variable-only model (*P* < 0.001).

**Conclusions:**

This study establishes a reproducible OCT radiomics pipeline and demonstrates the feasibility of applying unsupervised and supervised machine-learning approaches to pRNFL OCT data. Radiomic features show promising potential for improving the differentiation between MOGAD-ON and MS-ON.

**Financial Disclosure(s):**

Proprietary or commercial disclosure may be found in the Footnotes and Disclosures at the end of this article.

Optic neuritis (ON) is a common manifestation of multiple sclerosis (MS), affecting approximately 20% of patients with MS as an initial attack.^1^ A key alternative diagnosis, especially when clinical or magnetic resonance imaging (MRI) red flags are present, is myelin oligodendrocyte glycoprotein antibody–associated disease (MOGAD). Multiple sclerosis–associated ON (MS-ON) and MOGAD-associated ON (MOGAD-ON) share several clinical features, including acute painful vision loss and responsiveness to corticosteroid therapy. However, they differ in their typical radiological patterns and mostly in their relapse risk and their long-term visual prognosis. Myelin oligodendrocyte glycoprotein antibody–associated disease ON is characterized by more severe vision loss at nadir compared with MS-ON, often prompting a more intensive treatment, such as repeated high-dose intravenous corticosteroids and occasionally plasmapheresis.^2^, ^3^, ^4^ Although long-term visual prognosis is generally not worse in MOGAD-ON compared with MS-ON, it largely depends on the administration of appropriate treatment in a timely manner.^3^^,^^5^, ^6^, ^7^ For these reasons, identifying the underlying etiology is essential and should be achieved as early as possible.

The diagnosis of the underlying disease is generally based on brain and spinal cord MRI and on the detection of anti-MOG antibodies in serum or cerebrospinal fluid, according to current expert consensus recommendations and diagnostic criteria that are regularly updated.^8^^,^^9^ However, when MRI findings are unremarkable, and because antibody testing may require time to confirm serostatus, establishing the diagnosis during the acute phase of a first episode is not always possible. In such cases, OCT may provide valuable adjunctive information and assist in orienting the differential diagnosis. Studies have shown significantly increased peripapillary retinal nerve fiber layer (pRNFL) thickness in MOGAD-ON than MS-ON during the acute phase, followed by more marked optic disc atrophy in the chronic stage.^4^^,^^10^^,^^11^

Beyond conventional quantitative OCT metrics, emerging image analysis approaches offer the potential to uncover previously inaccessible information embedded within OCT images. Radiomics is a technique originally developed in the field of radiology that involves extracting a large number of features from images usually imperceptible to the human eye to reveal patterns that may aid in diagnosis.^12^ Radiomics enhances conventional image analysis through the automated, high-throughput extraction of numerous quantitative features from medical images. These features include first-order statistics describing intensity distributions, texture features, heterogeneity patterns, and shape-based descriptors characterizing the geometry and morphology of anatomical structures. The extracted radiomic features are subsequently analyzed using advanced statistical modeling and machine-learning techniques to identify clinically relevant associations and improve disease characterization.^12^ This approach, increasingly applied in radiology and nuclear medicine, particularly for tumor characterization, is only beginning to emerge in ophthalmology. Radiomics has been shown to be useful in evaluating atrophic age-related macular degeneration and in diabetic macular edema and age-related macular degeneration for characterizing “super-responders” to anti-VEGF therapy.^13^, ^14^, ^15^ It has also been applied to identify eyes with posterior segment inflammation^16^ and to reveal biomarkers of central serous chorioretinopathy.^17^ In the neuro-ophthalmology field, radiomics has been used in MS and neuromyelitis optica spectrum disorder, but exclusively on MRI images.^18^^,^^19^ Up to now, the application of radiomics to ON using OCT images has not been established.

The main objective of this study was to demonstrate the feasibility of an OCT-based radiomics approach in ON by exploring how far pRNFL OCT-derived radiomics features could help differentiate between MS-ON and MOGAD-ON.

## Methods

### Participants

Data were obtained from an existing cohort of patients followed at the Ophthalmology Department of the University Hospitals of Marseille, France. The Optic Neuropathies of la Timone cohort comprises longitudinal data from patients diagnosed with optic neuropathies of various etiologies, including neuroinflammatory causes (MS, myelin oligodendrocyte glycoprotein antibody–associated disease [MOGAD], neuromyelitis optica spectrum disorder, and sarcoidosis), as well as noninflammatory causes (e.g., ischemic or compressive optic neuropathies). For the present study, patients with ON associated with MS or MOGAD were extracted from the Optic Neuropathies of la Timone cohort and included in the analysis. Optic neuropathies were either inaugural episodes or recurrent events. The exclusion criterion was the presence of ophthalmic comorbidities that could cause retinal or optic damage, such as chronic open-angle or angle-closure glaucoma, optic atrophy of other etiology, or age-related macular degeneration.

### Ophthalmic Data

Clinical data were extracted, including demographic variables (age and sex), clinical characteristics, and laterality. OCT scans were performed with the Spectralis spectral-domain OCT (Heidelberg Engineering; pupils not dilated; eye tracking function on). Only OCT scans with a signal strength ≥20 dB were included in the analysis. For measurement of the pRNFL, we used a ring scan around the optic nerve head (12°, 1536 A-scans, 16 ≤ automatic real-time averaging ≤100) using the device-internal segmentation module 6.0.14.0. Scans not meeting the OSCAR-IB consensus criteria were excluded.^20^ Peripapillary retinal nerve fiber layer thickness measurements were obtained from the Heidelberg device. OCT images were then exported in E2E file format, a proprietary data format used by Heidelberg Engineering for storing OCT imaging data.

The E2E files were converted and organized following a structure inspired by the Brain Imaging Data Structure standard, commonly used in neuroimaging to enhance reproducibility, transparency, and multimodal data integration. A custom Python script (oct2bids.py, Python, v3.12) partly based on OCT-Converter^21^ was developed to automate data conversion and organization, enabling the extraction of OCT volumes, segmentation data, fundus images, and acquisition metadata (date, scan type, and eye laterality) into a standardized Brain Imaging Data Structure-like hierarchy.

Using a dedicated Python script (ring2mask.py), the segmentation contours stored in the E2E metadata were extracted to generate binary masks for the pRNFL, the underlying retina, and the entire retina, which were saved in Neuroimaging Informatics Technology Initiative (NIfTI) and Portable Network Graphics (PNG) formats for subsequent radiomic feature extraction.

### Extraction of Radiomics Features

Radiomic features were extracted using the open-source PyRadiomics library (3.1), a widely used tool in medical imaging.^22^ Features were computed from regions of interest defined by pRNFL masks on OCT images, with extraction parameters specified in .yaml configuration file tailored to OCT. Default settings were used in this study.

The extracted data were exported as .csv tables, with rows representing OCT acquisitions from either the right or left eye per session and columns representing radiomic features or clinical variables. Features with constant or null values were removed because they were noninformative. The data set was filtered to retain only eyes with inaugural ON or recurrent ON episodes in patients with a prior history of ON.

### Machine Learning

Machine-learning analyses were conducted using the scikit-learn library with Python 3.12 on Google Colab, following a standard machine-learning workflow.^23^

After several trials, recursive feature elimination was chosen, selecting the most informative features.

Unsupervised clustering was explored using K-means (2 clusters for MS and MOGAD), density-based spatial clustering of applications with noise (DBSCAN), and dimensionality reduction techniques (principal component analysis) for visualization. Clustering performance was assessed using the Silhouette score, which measures cluster cohesion and separation, and the adjusted Rand index (ARI), which compares the obtained clusters with the ground-truth MS/MOGAD labels.

Several supervised learning models, including logistic regression (logistic regression with L2 penalty) and linear support vector machine (SVM), were trained. Logistic regression and SVM were selected because they are low-variance linear models that are robust in small data sets and allow interpretability of the radiomic signature. Random Forest was not retained because of the risk of overfitting related to the cohort size and the presence of repeated measurements per patient. In a first step, clinically relevant variables were included in the model, namely age, laterality of ON, time from symptom onset to OCT acquisition, pRNFL thickness, and sex. A second model was then built using the 3 selected radiomic features alone. Finally, a combined model integrating both clinical and radiomic features was developed. A fivefold cross-validation scheme was implemented. At each iteration, 1 distinct subset of patients (20%) was held out for testing while the remaining 4 subsets (80%) were used for training, such that each patient contributed to the test set exactly once, and model performance was averaged across all 5 splits. Fivefold cross-validation was used to ensure that the entire cohort was iteratively used for both training and testing, thereby reducing the risk of performance estimates driven by a single random train–test partition.

Pooled performance measures were obtained by aggregating the predictions from all 5 test folds: area under the receiver operating characteristic curve (AUC), accuracy, F1-score, sensitivity, and specificity. Sensitivity and specificity were computed at the default decision threshold of 0.50 for all models. Area under the receiver operating characteristic curve comparisons were performed using pooled cross-validation predictions, obtained by assembling the predicted probabilities from each of the 5 test folds into a single vector (266 observations in total). Each patient contributed to the test set exactly once, ensuring that no data leakage occurred. The DeLong test was then used to statistically compare AUCs between models. Receiver operating characteristic curves were generated accordingly.

Overview of the radiomics processing and analysis pipeline is presented in Figure 1.

**Figure 1:**
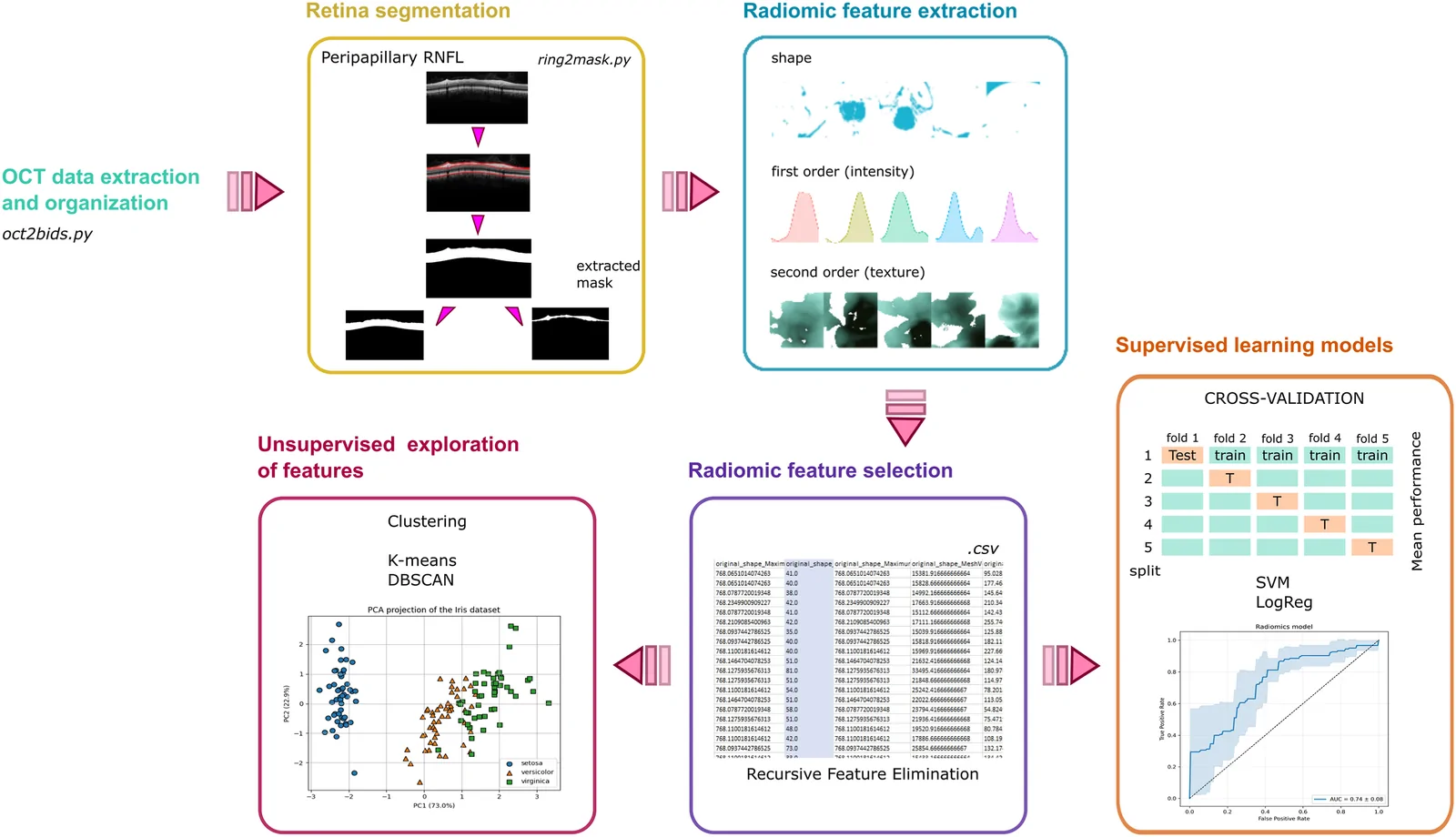
Overview of the radiomics processing and analysis pipeline. AUC = area under the receiver operating characteristic curve; DBSCAN = density-based spatial clustering of applications with noise; LogReg = logistic regression; RNFL = retinal nerve fiber layer; SVM = support vector machine.

### Ethics

The study adhered to the Declaration of Helsinki. Opt-out consent was obtained from the patients in the cohort, which was approved by the Hospital Review Board under reference number PADS24-71_dgr. Prior inclusion in the retrospective cohort implied consent for the use of the data in multiple retrospective analyses. The study has been approved by the Ethics Committee of the French Society of Ophthalmology (IRB00008855 Société Française d’Ophtalmologie IRB#1).

## Results

A total of 45 patients (61 eyes with ON) were included in the analysis, including 28 patients with MS and 17 patients with MOGAD. Almost all cases corresponded to first episodes of ON, with only 1 patient in the MOGAD group and 1 patient in the MS group with recurrent ON. Some patients had multiple examinations available. Overall, 266 examinations were included in the analysis. In the MOGAD group, 85.7% of OCT acquisitions were performed outside the acute phase (i.e., >1 month after symptom onset), compared with 74.4% in the MS group, with no significant difference in disease timing between groups. A total of 102 radiomic features were extracted and reduced to 62 after removal of null or constant variables. After feature selection using machine-learning methods, 3 variables were retained based on their stability across folds: *original_shape_MajorAxisLength* (same unit as the input image, μm), *original_shape_Maximum2DDiameterSlice* (same unit as the input image, μm), and *original_glszm_LargeAreaLowGrayLevelEmphasis* (normalized dimensionless value). The selected features predominantly describe lesion geometry (shape and size) and gray-level distribution heterogeneity (texture). Demographic characteristics, clinical characteristics of the patients, and selected radiomic features are presented in Table 1.

**Table 1:** Demographic Data, Clinical Data and Selected Radiomic Features in Patients with MS and Patients with MOGAD

*N* = 45 Patients (61 Eyes, 266 Examinations)	MS (*N* = 28, 33 Eyes, 110 Examinations)	MOGAD (*N* = 17, 28 Eyes, 156 Examinations)
Sex, *n* (%)		
Female	19 (67.9%)	9 (52.9%)
Age at baseline (median [IQR])	29 (9.2)	30 (14.1)
Affected eye, n (%)		
- Bilateral	5 (17.9%)	11 (64.7%)
- Unilateral: right eye	14 (50%)	0
- Unilateral: left eye	9 (32.1%)	6 (35.3%)
Number of sessions per patient (mean ± SD)	3.18 ± 2.7	5.47 ± 3.99
Radiomic features (mean ± SD)		
- original_shape_MajorAxisLength	827.47 ± 35.43	864.58 ± 39.76
- original_shape_Maximum2DDiameterSlice	46.95 ± 10.49	42.32 ± 15.91
- original_glszm_LargeAreaLowGrayLevelEmphasis	320 663 725 ± 205 403 120	308 661 536 ± 365 316 279

GLSZM = gray-level size zone matrix; IQR = interquartile range; MS = multiple sclerosis; MOGAD = myelin oligodendrocyte glycoprotein antibody–associated disease; SD = standard deviation.

Results for the supervised classification models are presented below. Table S1 (available at www.ophthalmologyscience.org) presents the number of subjects and group distribution across the different folds. Pooled performance metrics from the 2 models on the test set using cross-validation are summarized in Table 2. Both models achieved higher AUC values when trained on radiomic features compared with clinical variables alone (namely age, laterality of ON, time from symptom onset to OCT acquisition, pRNFL thickness, and sex). The superiority of radiomics and combined models over the clinical model alone was statistically significant for both algorithms (Table 3). The combined model yielded a numerically higher AUC than radiomics alone, but without statistical significance. The receiver operating characteristic curves illustrating the performance of the logistic regression and SVM models are presented in Figure 2.

**Table 2:** Pooled Performance Metrics of the Supervised Classification Models on the Test Set. Metrics Include AUC, Accuracy, F1-Score, Sensitivity, Specificity

	Model	AUC	Accuracy	F1	Sensitivity	Specificity
Clinical	LogReg	0.584	0.695	0.743	0.750	0.618
	SVM	0.547	0.733	0.782	0.814	0.618
Radiomics	LogReg	0.763	0.695	0.720	0.667	0.736
	SVM	0.699	0.680	0.735	0.756	0.573
Combined	LogReg	0.788	0.729	0.772	0.782	0.655
	SVM	0.753	0.699	0.760	0.814	0.536

AUC = area under the receiver operating characteristic curve; LogReg = logistic regression; SVM = support vector machine.

**Table 3:** Comparison of AUC Between Models Using the DeLong Test

Comparison	Model	AUC 1	AUC 2	*Z*	*P* Value
Radiomics vs. clinical	LogReg	0.763	0.584	3.19	0.001∗
Combined vs. clinical	Log Reg	0.788	0.584	5.77	<0.001∗
Combined vs. radiomics	LogReg	0.788	0.763	0.91	0.361
Radiomics vs. clinical	SVM	0.699	0.547	2.63	0.008∗
Combined vs. clinical	SVM	0.753	0.547	5.68	<0.001∗
Combined vs. radiomics	SVM	0.753	0.699	1.83	0.068

AUC = area under the receiver operating characteristic curve; LogReg = logistic regression; SVM = support vector machine.

∗ Statistically significant (P < 0.05).

**Figure 2:**
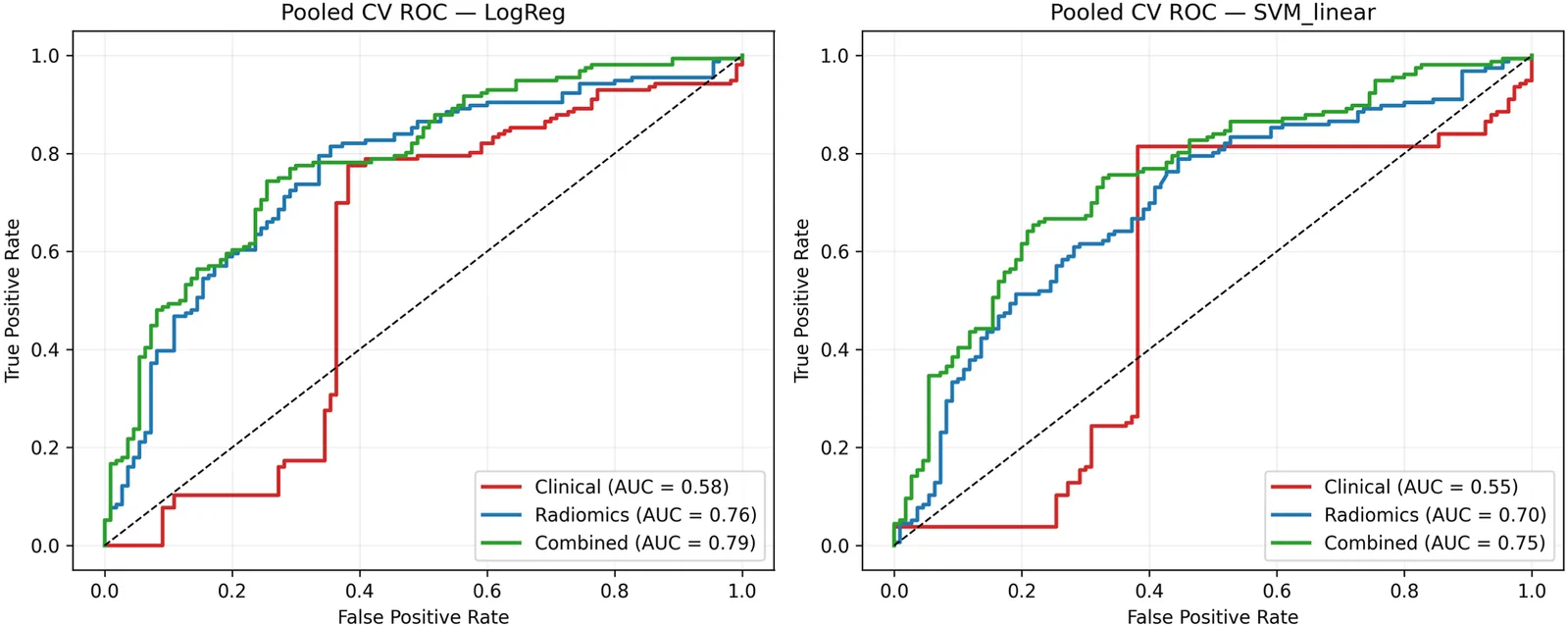
Pooled cross-validation (CV) receiver operating characteristic (ROC) curves of the logistic regression (LogReg) and support vector machine (SVM) models. AUC = area under the receiver operating characteristic curve.

Unsupervised clustering demonstrated limited ability to detect internal structure within the 3-feature radiomic space. The K-means algorithm yielded a Silhouette score of 0.598 and an ARI of –0.005. Density-based spatial clustering of applications with noise identified 2 clusters, with a Silhouette score of 0.494 and an ARI of –0.013, with 26 noise outliers.

## Discussion

In this proof-of-concept study, the models were designed to differentiate MS from MOGAD using peripapillary OCT-derived radiomic features across different stages of ON evolution and including both inaugural and recurrent episodes. Results suggest that supervised classification models performed significantly better when using radiomic features than when relying on clinical variables alone. The combined model significantly outperformed the clinical-variable-only model. The combined model yielded a numerically higher AUC than radiomics alone, but without statistical significance. These results are encouraging, given the complexity of the clinical context.

Together, the 3 selected radiomic features characterize lesion morphology and texture, potentially reflecting differences in underlying pathological processes. Size-related features are clinically interpretable. The variable *original_shape_MajorAxisLength* quantifies the length of the longest axis of the fitted ellipsoid to the segmented region, thereby reflecting the primary spatial extent of the region of interest, namely the RNFL, and reflecting changes in RNFL shape or spatial extent. This feature was lower in patients with MS, which may be consistent with optic atrophy secondary to previous episodes of ON and retrograde degeneration observed in patients with MS after several years of disease evolution. *Original_shape_Maximum2DDiameterSlice* measures the largest Euclidean distance between any 2 points within the region of interest on the same axial slice, providing a complementary descriptor of maximal in-plane spread that is less dependent on global 3D assumptions. In contrast, the texture-related feature *original_glszm_LargeAreaLowGrayLevelEmphasis* is currently difficult to interpret in light of existing clinical knowledge. It is a texture feature derived from the gray-level size zone matrix, which emphasizes the presence of large homogeneous zones with low intensity values, thus capturing the extent of spatially extensive low-signal areas within the region of interest. This feature could capture subtle tissue characteristics not readily discernible by the human eye. Overall, the combination of these features appears to carry a relevant signal. This radiomic signature may therefore represent a specific fingerprint associated with a distinct subtype of inflammatory pathology of the central nervous system.

Previous studies have demonstrated distinct pRNFL dynamics between MOGAD-ON and MS-ON. During the acute phase, MOGAD-ON is characterized by a greater increase in pRNFL thickness compared with MS-ON, reflecting more pronounced optic disc edema.^10^ During the chronic phase, several studies have shown a more pronounced pRNFL atrophy in MOGAD-ON than in MS-ON.^2^^,^^4^^,^^11^ Myelin oligodendrocyte glycoprotein antibody–associated disease ON is associated with diffuse global pRNFL thinning across all quadrants, whereas MS-ON typically exhibits a more selective pattern, predominantly affecting the temporal quadrant, with relatively preserved global thickness.^2^^,^^4^^,^^11^ Quantitatively, global pRNFL values after a single ON episode are significantly lower in MOGAD-ON than in MS-ON in both adult and pediatric cohorts, highlighting disease-specific patterns of neuroaxonal damage.^4^^,^^11^

Although conventional OCT metrics such as global and sectoral pRNFL thickness provide valuable structural information, they may not fully capture the complexity of disease-specific microstructural patterns. In this context, OCT-based radiomics offers a complementary approach by extracting high-dimensional features beyond mean thickness and is increasingly applied across medical imaging fields beyond radiology. Radiomics approaches have been increasingly applied to OCT imaging across a wide range of retinal diseases, demonstrating their ability to capture clinically meaningful information beyond conventional thickness-based metrics. In macular disorders, OCT-derived radiomic features have shown value in characterizing disease severity and progression, particularly in age-related macular degeneration and diabetic macular edema, where they have been associated with treatment response, durability, and the identification of so-called “super-responders” to anti-VEGF therapy.^13^, ^14^, ^15^ Beyond therapeutic stratification, radiomics has also been used to detect subtle inflammatory changes in the posterior segment that may not be readily apparent on standard OCT interpretation.^16^ More recently, radiomics-based OCT analyses of the choroid have revealed disease-specific biomarkers in central serous chorioretinopathy, further supporting the versatility of this approach across different retinal and choroidal pathologies.^17^ In contrast, applications of radiomics in neuro-ophthalmology have so far been largely confined to MRI, particularly in MS and neuromyelitis optica spectrum disorder, where radiomic features of the optic nerve and brain have been explored as markers of tissue damage and functional outcome. Despite the widespread clinical use of OCT in ON and its well-established role in assessing neuroaxonal loss, the application of radiomics to ON using OCT images has, up to now, not been established.

This study shows the feasibility of an OCT-based radiomics approach in optic neuropathies. Beyond feasibility, its potential clinical relevance is substantial. In practice, the extraction of radiomic features is rapid and could reasonably be envisioned as being integrated into OCT devices in the near future. In contrast to large deep learning models applied to entire image data sets, which are computationally intensive, time-consuming, and require substantial processing power, our radiomic feature processing approach is faster and easier to integrate into clinical practice. Such applicability may have a direct impact on clinical practice, particularly in the context of emerging disease-modifying therapies currently under investigation in ON, as reflected by multiple ongoing trials such as the MOGWAI study.^24^, ^25^, ^26^, ^27^

This study has several limitations. First, the relatively small sample size constrained the use of complex machine-learning models and limited the number of features that could be reliably incorporated. As a result, simple models were deliberately favored to reduce the risk of overfitting. Although this limitation reflects the rarity of ON (annual incidence around 4/100 000^28^), particularly MOGAD-ON (5% of ON^28^), it also highlights the need for multicenter cohorts. Increasing the number of patients in future studies will be essential to improve model robustness and potentially allow the inclusion of more informative features. Second, variability in OCT acquisition timing and the inclusion of repeated OCT scans without longitudinal modeling may have introduced temporal noise. However, a patient-level split was performed to prevent temporal leakage. Additionally, the distribution of OCT acquisitions performed during the acute phase and outside the acute phase (defined as more than 1 month after symptom onset) was comparable between the MS and MOGAD groups. Nevertheless, the model was able to discriminate between the 2 pathologies despite this potential temporal heterogeneity. In future studies with larger data sets, restricting analyses to OCT examinations performed either in the first month after the first episode of ON or at least 6 months after the first episode of ON may help reduce temporality-related variability. Third, a slight class imbalance between patients with MS and MOGAD may have influenced model performance and algorithm selection. The methodological approach was intentionally designed to be reproducible and well suited to a limited sample size. Automatic feature selection using recursive feature elimination helped mitigate overfitting while preserving interpretability. The choice to retain 3 features was a trade-off between model complexity and stability. This parameter could be adjusted in future work, for example, by retaining 4 or 5 features to capture additional variance. Future studies should test these approaches on images from alternative OCT devices and imaging platforms to assess their robustness and generalizability.

The use and interpretation of unsupervised learning approaches raise important methodological considerations because they require expertise in machine learning and careful model selection to ensure biological interpretability. More specifically, the choice of unsupervised clustering method influences the apparent structure of the data. Although K-means enforces a binary partition aligned with the clinical question, DBSCAN allows for more flexible cluster shapes and the explicit identification of noise, which may better reflect the underlying biological complexity of ON. The presence of unassigned observations identified as noise by DBSCAN likely reflects the biological and temporal heterogeneity of ON. These examinations may correspond to intermediate or atypical radiomic profiles, influenced by disease stage, relapse history, or interindividual variability.

The present findings provide a foundation for future developments. Increasing the sample size remains a key objective and will be pursued through continued patient inclusion as well as multicenter and international collaborations. In this context, the development of open-source, standardized pipelines for OCT preprocessing, feature extraction, and analysis, as presented in this study, will ensure reproducibility and facilitate data harmonization across centers. Importantly, the inclusion of well-characterized healthy control subjects will be essential, both for supervised and unsupervised approaches, to provide robust reference distributions and improve model interpretability. Future studies should also incorporate longitudinal designs to better capture temporal changes and disease dynamics. Extending this approach to macular OCT, by integrating not only the RNFL but also the ganglion cell layer, may provide complementary and potentially more sensitive markers of neuroaxonal damage. Moreover, full retinal layer segmentation would enable a more comprehensive assessment across all relevant structures, including the ganglion cell inner plexiform layer, as well as the inner nuclear layer, the outer nuclear layer, and the inner plexiform layer alone, which could show early changes related to synaptopathy. In addition, macular acquisitions typically consist of volumes comprising 19 or 21 B-scans, thereby increasing the number of images available for analysis and potentially enhancing feature extraction robustness and allowing the inclusion of a larger number of radiomic features in the model. Furthermore, the use of deep learning models may allow the capture of more complex and nonlinear relationships within high-dimensional OCT-derived feature spaces. Finally, as larger data sets become available, increasing the number of radiomic features included in the models will allow more variance to be captured and help avoid overfitting. In the long term, this approach could be applied to differentiate among a broader spectrum of optic neuropathy etiologies.

## Conclusion

In conclusion, this study establishes a reproducible OCT data and radiomic feature extraction pipeline. This proof-of-concept study shows that radiomic analysis of peripapillary OCT outperforms clinical examination in differentiating MS-ON from MOGAD-ON, despite a limited sample size. Simple and interpretable machine-learning models show promising discriminative performance, supporting the potential clinical relevance of this approach.

## Acknowledgments

The authors gratefully acknowledge the support of the CMA CGM Health Chair (AMX-24-COD-003), established in partnership with CMA CGM, Aix-Marseille Université, and the AMidex Foundation. The authors also acknowledge the Excellence Initiative of Aix-Marseille University (AMIDEX), a French “Investissements d’Avenir” programme (AMX-19-IET-002), as well as Marseille Imaging, Neuromyelitis Optica Study Group—Nemos, and all collaborating partners for their valuable scientific, technical, and logistical support.
